# P-442. The Contemporary Spectrum of Haemophilus influenzae Disease in Children: An Active Surveillance Study

**DOI:** 10.1093/ofid/ofaf695.657

**Published:** 2026-01-11

**Authors:** Jonathon C McNeil, Melinda Benavides, Meghan Walther, Lauren M Sommer, Kristina G Hulten

**Affiliations:** Baylor College of Medicine, Houston, TX; Baylor College of Medicine, Houston, TX; Baylor College of Medicine, Houston, TX; Baylor College of Medicine, Houston, TX; Baylor College of Medicine, Houston, TX

## Abstract

**Background:**

Significant shifts in the epidemiology of respiratory pathogens in children have occurred in the post-SARS-CoV-2 era. In a retrospective study, we recently reported an increase in invasive *Haemophilus influenzae* (Hi) disease in children, largely driven by non-typeable strains (NTHi). We reassessed the epidemiology of Hi disease in children in a prospective surveillance study.

**Figure 1.**
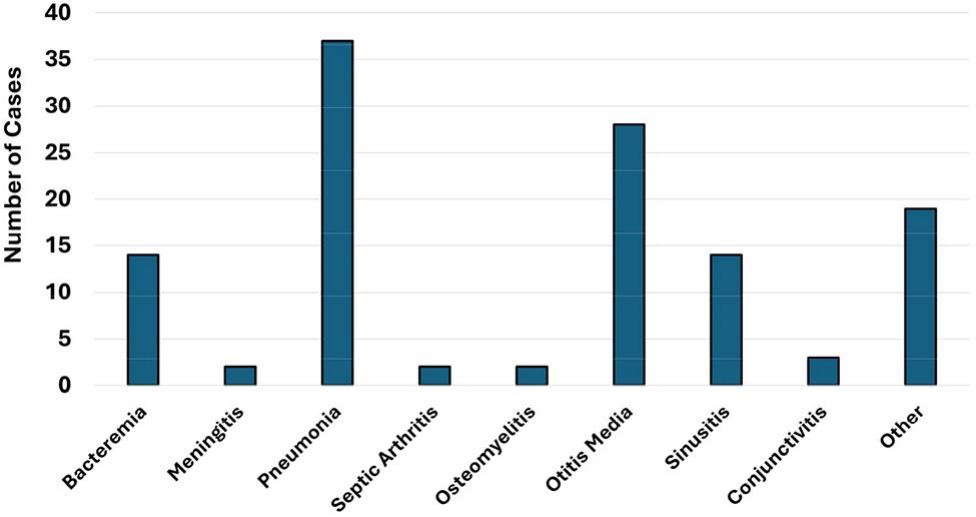
Haemophilus influenzae Diagnoses Number of H. influenzae cases with each clinical diagnosis

**Figure 2.**
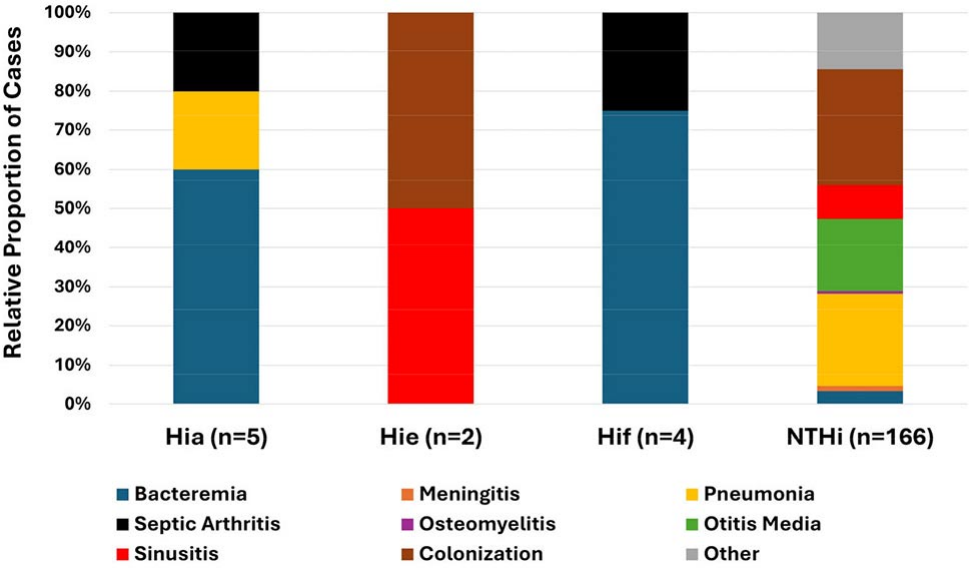
Capsular Type and Disease Manifestation The relative proportion of each clinical diagnosis by capsular type. Hia- Haemophilus influenzae type a; Hie- H. influenzae type e; Hif- H. influenzae type f; NTHi- nontypeable H. influenzae.

**Methods:**

Hi isolates identified by the clinical microbiology laboratory of Texas Children’s Hospital from Nov 2023-Dec 2024 were captured. All isolates underwent capsular typing using a multiplex PCR assay. Medical records were reviewed. Cases with isolation of Hi from a non-sterile site without accompanying signs/symptoms of infection were considered colonization. Invasive infections were considered those with isolation of Hi from a sterile site or a respiratory source with a clinical diagnosis of pneumonia.

**Figure 3.**
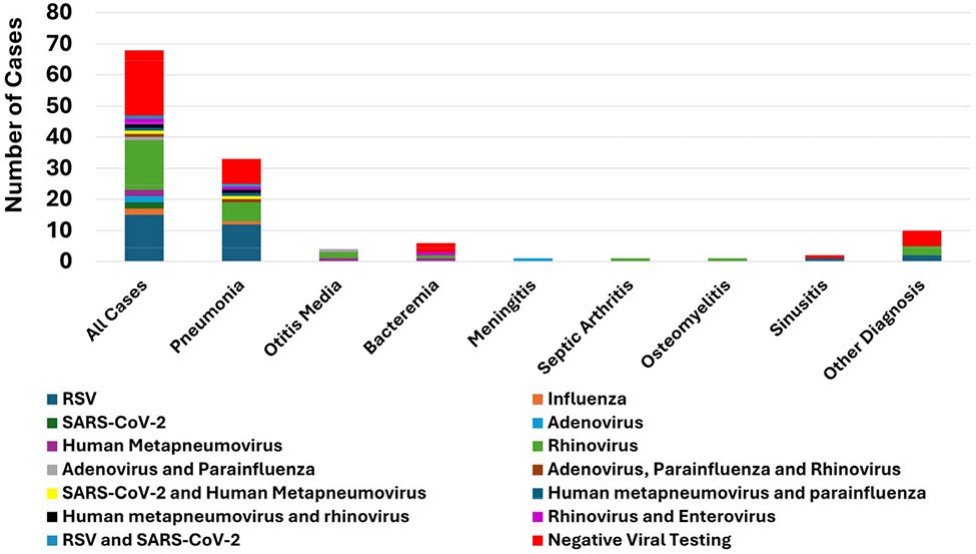
H. influenzae and Respiratory Viruses Frequency of respiratory virus codetection (y-axis) with each H. influenzae diagnosis

**Results:**

193 Hi isolates were obtained from 178 children; the median age of subjects was 2.9 years (IQR: 1.4-6.7 years). 23.8% of isolates were colonizing. Among infections, the most common diagnoses were pneumonia (28.6%), otitis media (22.2%) and bacteremia (9.5%, Figure 1); 46% of cases were associated with an invasive infection. The most common capsular types were NTHi (93%) and type a (Hia, 2.9%). Overall, encapsulated strains comprised 11 cases. The majority of Hi pneumonia were NTHi (97.3%) and 91% were admitted to the ICU. Of those cases with otitis media, 71.4% presented with otorrhea; co-pathogens were identified in 42.8%, most commonly *Streptococcus pneumoniae*, *Staphylococcus aureus* and *Moraxella*. Bacteremia was the most common manifestation of Hia (60%) and type f (Hif, 75%, p< 0.001, Figure 2). Among subjects with testing performed, a respiratory virus was co-detected in 70.4%, most commonly RSV and rhinovirus (Figure 3); viruses were more often detected in invasive infections (93.5% vs. 52.5%, p< 0.001).

**Conclusion:**

Hi contributes to significant morbidity in children with the most common manifestation being NTHi pneumonia. While encapsulated strains are relatively rare, these organisms are disproportionately associated with invasive disease including bacteremia. Invasive Hi in children is frequently associated with respiratory viruses.

**Disclosures:**

Jonathon C. McNeil, MD, Merck, Sharp and Dohme: Grant/Research Support|UpToDate: Royalties Kristina G. Hulten, PhD, Pfizer: Grant/Research Support

